# Biologic TNF inhibiting agents for treatment of rheumatoid arthritis: persistence and dosing patterns in Germany

**DOI:** 10.1186/s13561-014-0032-4

**Published:** 2014-11-23

**Authors:** Sarah Neubauer, Mary Cifaldi, Thomas Mittendorf, Arijit Ganguli, Malte Wolff, Jan Zeidler

**Affiliations:** Center for Health Economics Research Hannover (CHERH), Leibniz Universität Hannover, Otto-Brenner-Straße 1, D-30159 Hannover, Germany; AbbVie Inc, North Chicago, IL USA; Herescon GmbH, Hannover, Germany

**Keywords:** Rheumatoid arthritis, Claims data, Persistence, Dosing patterns, Germany, Tumor necrosis factor inhibitor

## Abstract

**Objective:**

To obtain detailed real-world data on persistence and dosing patterns in the utilisation of the TNF inhibitors adalimumab, etanercept, and infliximab in rheumatoid arthritis (RA) patients treated in Germany.

**Methods:**

In this retrospective observational study claims data of a major German health insurance fund between 2005 and 2008 were analysed. Patients receiving at least one prescription of adalimumab, etanercept or infliximab were identified and categorised as “TNF inhibitor naive" or “TNF inhibitor continuing”. For the calculation of TNF inhibitor persistence a survival analysis with the Kaplan–Meier estimator was used. A Cox regression was used to analyse, if any relevant factors were influencing persistence. Dosage increase rates were analysed for adalimumab, etanercept and infliximab. Sensitivity analyses based on variations in gap length were conducted.

**Results:**

A total of 2,201 RA patients were identified. 1,468 of these patients were TNF inhibitor naive patients and 733 were defined as TNF inhibitor continuing patients. There were no significant differences in the treatment persistence rates between adalimumab, etanercept and infliximab for TNF inhibitor naive and continuing patients. The persistence rate after three years was 22.47% for adalimumab, 24.27% for etanercept and 21.49% for infliximab naive patients. For continuing patients, the persistence rate after three years was 32.88% for adalimumab, 30.95% for etanercept, and 33.90% for infliximab, respectively. Gender, medication and Charlson Comorbidities Index did not influence the persistence significantly. Dosage increase occurred in 7.3% adalimumab, 1.4% etanercept, and 17.2% infliximab naive patients and 5.8%, 1.1% and 11.9% respectively in the continuing patients.

**Conclusions:**

In this study, there were no significant differences in persistence among adalimumab, etanercept and infliximab treated patients. Consistent with previous research, there was a higher dose escalation for infliximab than for the two subcutaneous treatments, adalimumab or etanercept.

## Background

Rheumatoid arthritis (RA) is defined as a systemic autoimmune disease which is characterised by chronic progression of joint damage. RA is known as the most frequent chronic inflammatory disease of the joints. Its prevalence has been estimated in a range between 0.5% and 1% for different populations worldwide [1]. From the economic point of view studies have shown that RA is associated with a high economic burden [2,3].

The combination of disease-modifying anti-rheumatic drugs (DMARDs) and the development of tumor necrosis factor (TNF) inhibitors have for the first time lead to a clinical remission of RA and induce a delay or complete stop of the clinical and radiological progression of the disease, thus improving the quality of life of many patients with RA [4]. Therefore, TNF inhibitors comprise an important part of current treatment recommendations in Germany and other countries [5–7]. The first TNF inhibitors which have been approved for application in RA treatment in Germany were adalimumab, etanercept, and infliximab.

In our study we investigate the issues of persistence and dosing patterns of TNF inhibitors in Germany. There have been conducted a number of studies in this field. Carmona and Gomez-Reino studied TNF inhibitors for different time periods (one, two and three years) based on a Spanish population [8]. They report persistence rates of 83% (CI: 81–84), 72% (CI: 71–74) and 65% (CI: 63–67) respectively. A 5 years follow-up Dutch study reports estimates for the cumulative persistence of 70% after the first year with a decrease of the rate to 45% after the last year [9]. Jobanputra et al. reported from a pragmatic randomized trial of adalimumab versus etanercept on the primary endpoint of treatment discontinuation that no statistical significant difference on persistence was found although adalimumab had numerically higher persistence rates after one and two years of treatment 65.0% (58.3%) for adalimumab vs. 56.7% (43.3%) for etanercept at week 52 (week 104) [10]. The longest survey for infliximab is a seven years follow-up study conducted in Greece [11]. The authors estimate a persistence rate for infliximab after the first year of the treatment of 83%; they also report a decrease of the rate to 33% after seven years. For etanercept a persistence rate of 70% was estimated after the first year of the treatment, and after four years the rate remained at the level of 60%. Adalimumab showed a persistence rate of 84%, which declined to 45% after five years. Cho et al. estimate persistence rates for TNF inhibitors based on a Korean population [12]. They report an overall persistence rate of 73% for the period of twelve months and 61% for 18 months. Particularly for adalimumab and etanercept for the period of six months the rates were of 82% and 85% and for twelve months 73% and 78%, respectively. Another study from Switzerland indicates that infliximab is associated with higher overall discontinuation rates compared with adalimumab and etanercept, explaining it mainly with an increased risk of infusion or allergic reactions [13].

There is no strong evidence about persistence and dosing patterns of adalimumab, etanercept and infliximab in Germany. Our literature overview resulted only in one study provided by Zink et al. which analysed the TNF inhibitor persistence rates for the German health care system [14]. They estimate the persistence rate of etanercept and infliximab after a one year of 69% (CI: 62–75) and 65% (CI: 58–73), respectively. However, they exclude adalimumab from the analysis justifying this by a later start of enrolment on the German market. In our study we provide an analysis of persistence rates as well as for dosage modifications for all three TNF inhibitors for Germany. We base our study on claims data provided by a major health insurance fund in Germany over a four years observation period. One of the advantages of our analysis is the consideration of RA patients treated by all medical professionals [15], compared to Zink et al., who limit the analysis on patients treated by rheumatologists [14]. Therefore, our data could demonstrate the treatment patterns of all RA patients, even those who were not treated continuously by a rheumatologist. Using claims data for the analysis brings further advantages to our research. Compared with general clinical studies, claims data provide a better sampling reflecting real-world treatment patterns and avoids possible selection bias. The results of our study provide a considerable contribution to the research of the usage TNF inhibitors for RA treatment in Germany.

## Methods

### Data source

In order to address the study question we retrieved nationwide claims data for the period from 2005 to 2008. The dataset contains general individual information on a per-patient basis e.g. on age and gender as well as detailed data on diagnostic codes and prescribed medications including number of drug-packages. Moreover, the admission and discharge dates for inpatient cases, delivery and prescription date of medicinal products such as the frequency and the type of different therapies are available in the data. However, according to the German law information on clinical outcomes are not to be provided in the claims data set. The variables on the treatment in the dataset are grouped according to the relevant medical care: outpatient care, inpatient care, rehabilitation, medications. The study was designed from the point of view of a major German health insurance fund with nearly 6.5 million covered lives (DAK) in 2008. Based on this nationwide claims data base all RA-patients receiving at least one prescription of adalimumab, etanercept, or infliximab during the years 2005–2008 were identified.

### Study population and study design

**Figure 1:**
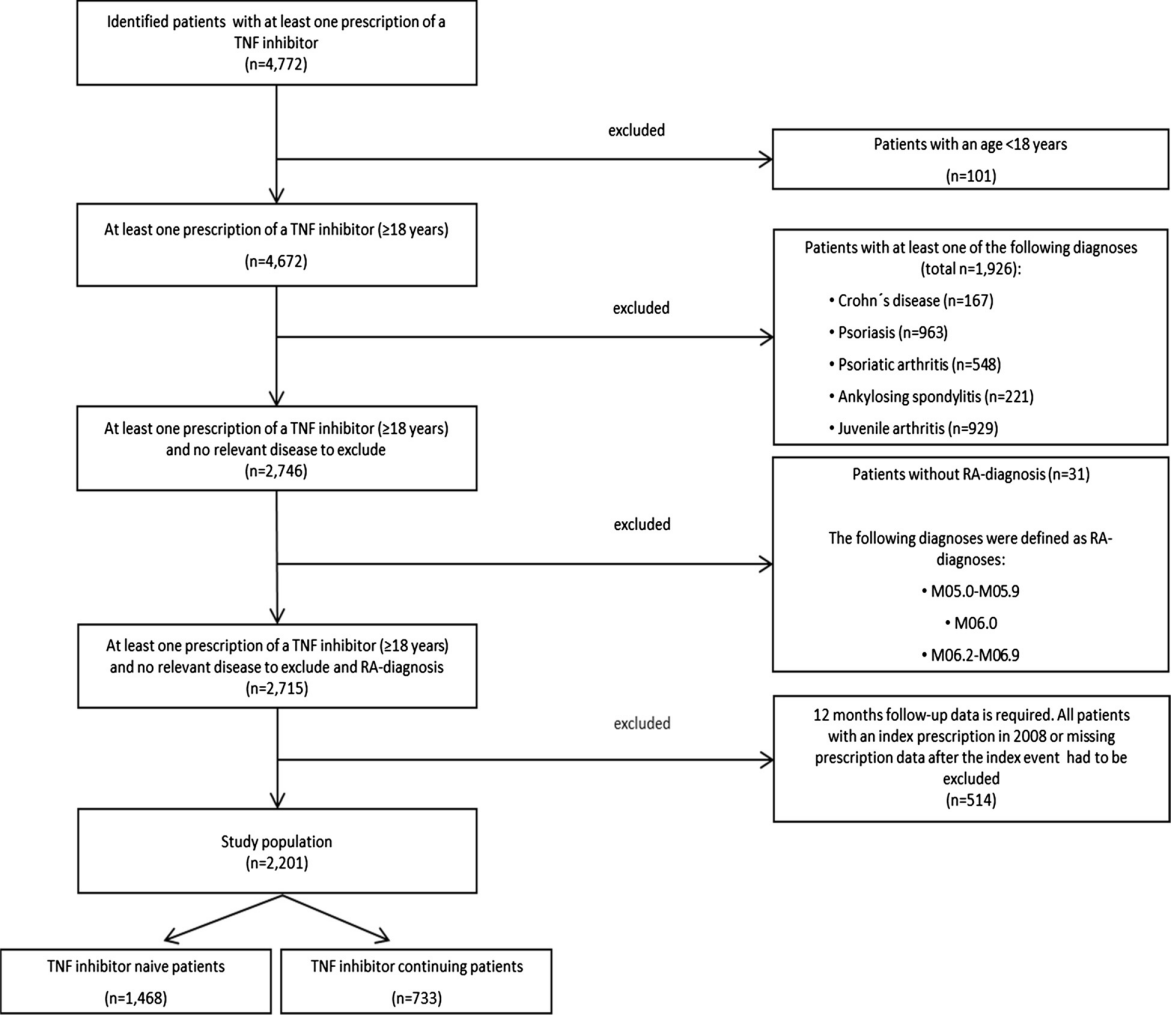
**Study population.**

The identification of the study population was based on the International classification of diseases, 10th revision (ICD-10) diagnoses codes and prescription data. All patients who had at least two secured diagnoses of rheumatic diseases or diseases of the connective tissue in the outpatient sector as well as at least one prescription of a TNF inhibitor during the study period 2005–2008 were initially included in the study. All included patients needed to be continuously insured in the specific health insurance fund between the years 2005–2008. From this initial population all patients were excluded who were younger than 18 years of age. Additionally, all patients with at least one diagnosis of an inflammatory disease like Crohn´s disease as well as patients without an RA-diagnosis were excluded. The reason for this is that TNF inhibitors can also be prescribed in other indications which are not in the focus of this study. In a last step all patients were excluded who had not a follow-up period of at least twelve months during study period (Figure 1).

**Figure 2:**
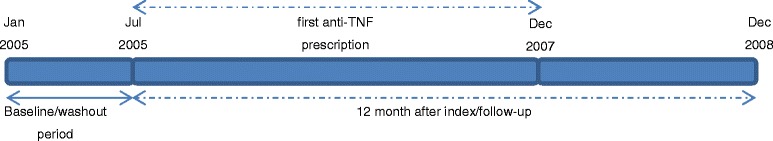
**Study period.**

Patients were categorized as “TNF inhibitor naive" or “TNF inhibitor continuing”. Naive patients were defined as individuals with RA who stated in the data no TNF inhibitor prescription during the baseline period of the study (January 2005 – June 2005). Continuing patients are defined as patients who had at least one claim for a TNF inhibitor during the baseline period and at least one claim in the follow-up period. For each patient in the final sample an index event was defined individually as the date of the first anti-TNF prescription after the baseline period (Figure 2).

For TNF inhibitor continuing patients all secured diagnoses in the baseline period (1st and 2nd quarter of 2005) were included for the analysis of patient characteristics. For TNF inhibitor naive patients the six months preceding the index event were defined as the period for the analysis of patient characteristics and all secured diagnoses of the first and the second quarter before their index quarter were included because outpatient diagnosis data were only available quarterly for Germany. The Bonferroni and Tukey-Kramer method were used to compare variables like gender, mean age and Charlson-Index between the TNF inhibitor patients. The Tukey-Kramer method is a multiple comparison test and it is used to determine whether three or more means differ significantly in an analysis of variance.

We retrieved the data on age at index date, gender, comorbidities, prescribed medication and other variables related to the treatment. Inpatient and outpatient data on diagnosis (ICD-10 codes) were used to measure the Charlson comorbidity index (CCI), which was defined as a sum of the weights related to each condition for which a patient had claims data available. The ICD-10 Coding Algorithms for Charlson Comorbidities based on Quan et al. [16] includes 17 comorbidities (myocardial infarction, congestive heart failure, peripheral vascular disease, cerebrovascular disease, dementia, chronic pulmonary disease, rheumatic disease, peptic ulcer disease, mild liver disease, diabetes without chronic complication, diabetes with chronic complication, hemiplegia or paraplegia, renal disease, any malignancy, including lymphoma and leukaemia, except malignant neoplasm of skin, moderate or severe liver disease, metastatic solid tumour, AIDS/HIV).

### Analysis of persistence

We define persistence as a period of time during which the patients receive a particular TNF inhibitor. We calculated it based on the index date and the date of the break in the treatment with the medication. Regarding to Wu et al. a “medication discontinuation” was defined as the first appearance of a gap in the index medication of more than 60 days [17]. The observation period ended at 31st December 2008. This date was set as the censoring date for the analysis under the condition that the break in the treatment did not exceed 60 days [17,18]. For adalimumab and etanercept we assume that discontinuation takes place when the gap between the perceived end date of a prescription as per label information (e.g. prescription date +14 days for a single adalimumab prescription) and the date of the following prescription was more than 60 days apart. For naive infliximab patients, we distinguish three possibilities when discontinuation occurs. A discontinuation was assumed when the gap between the first and the second infusion was a) more than 14 plus 60 days, b) more than 28 plus 60 days between the second and third infusion, and c) when the gap between subsequent infusions was more than 56 plus 60 days. A discontinuation for TNF inhibitor continuing patients was assumed when the gap between each infusion was more than 56 plus 60 days (for infliximab). Patients with only one prescription of TNF inhibitor were excluded in the analyses.

The ability to switch to another TNF inhibitor could be a major reason for a high discontinuation rate. Therefore, we analysed patients who did and who did not switch the TNF inhibitor during their treatment and measured the discontinuation rates. This is important to investigate the impact of switching e.g. due to side effects of the first medication. In addition, we provide sensitivity analyses varying the gap length. Therefore, the gap between two consecutive TNF inhibitor prescriptions was set at 30 and 120 days. This represents 50% and 200% of the 60 day gap as defined in the basic model. German claims data contains no information about medications prescribed during inpatient episodes. Therefore, the number of patients with at least one hospitalization was also analysed within a sensitivity analysis, as these patients might have received a TNF inhibitor application during that time.

The Kaplan–Meier estimator [19] was used for the calculation of TNF inhibitor persistence. In general, it estimates the survival function from life-time data. In other words the estimator is used to estimate the probability that a particular event does not occur for a test object in a specific time interval. In many clinical trials it is often used to measure the efficacy of an intervention based on the time until occurrence of a particular event. The overall probability of surviving at a given time is represented as a product of the conditional probabilities [20]. To compare the curves describing persistence of the three anti-TNFs the log-rank test was used.

Additionally a Cox regression was used to analyse if age, gender, persistence, type of TNF inhibitor, or comorbidities were influencing factors on persistence.

### Analysis of dosage increase rates

Adalimumab, etanercept, and infliximab are available in different administration forms (infusion, finished drug injection, syringe, and injector) and different dosages. The recommended dosage for etanercept is 25 mg twice a week or 50 mg once weekly. Adalimumab is recommended at a dosage of 40 mg every other week. In monotherapy some patients who experience a decrease in their response may benefit from an increase in dose to 40 mg adalimumab every week. Infliximab is administrated intravenously with a recommended dose of 3 mg per kg of body weight for RA. Additional infusions are administered two and six weeks after the initial infusion and at eight-week intervals thereafter. In addition, the dosing can be increased and the infusion intervals shortened depending on patient needs or other circumstances.

For every single prescription a specific calculation was made regarding the time horizon for which the prescription should suffice in accordance with the information given in the label of the prescription. The calculation was done assuming that the treatment was initiated as per the recommended dose. For instance a prescription of one syringe of adalimumab was assumed to last for 14 days. Since the dosing of infliximab depends on the patient´s body weight, which was not available in the database, the dose administered in the third infusion (i.e., associated with the third claim) was taken as the reference dosage for the analysis. The percentage of patients prescribed with a dose of more than the recommended dosage was calculated based on the approach suggested by Wu et al. [17]. The weekly dosage for each prescription was calculated as:

- Quantity multiplied by 7 and divided by the prescription gap (for adalimumab)
- Dosage multiplied by quantity multiplied by 7 and divided by the prescription gap (for etanercept)
- Number of vials multiplied by 7 and divided by the prescription gap (for infliximab)

A prescription gap was defined as the number of days between a TNF inhibitor prescription and the subsequent prescription. The average dosage within the first year of the treatment was compared with the recommended dosage (e.g. 0.5 syringes per week for adalimumab). An increase in dosage was defined as an observed average weekly dosing that exceeded the recommended dose for etanercept and adalimumab or the reference dose for infliximab by at least one third (e.g. an average of more than 0.66 syringes per week of adalimumab is defined as increase in dosage).

### Software and data protection

Data management and statistical analyses were realized with Microsoft® Access 2007 and Microsoft® Excel 2007. Additionally IBM SPSS Statistics version 19 and Stata version 11 were used for specific statistical analyses. The data available for analysis was in a de-identified form.

## Results

### Study population

A total of 2,201 patients were identified (Figure 1). 1,468 of these patients were defined as TNF inhibitor naive patients and 733 were defined as TNF inhibitor continuing patients. Mean age of the TNF inhibitor naive patients was 58 years (±12.00) with 88% being female. Mean age of the TNF inhibitor continuing patients was 55 years (±12.32) with 87% being female (Table 1). No significant differences could be observed in the age and gender distribution between subgroups.

**Table 1:** **Number of patients, demographic data and Charlson comorbidities at index**

	**TNF inhibitor naive patients (n = 1,468)**			**TNF inhibitor continuing patients (n = 733)**			**TNF inhibitor total patients (n = 2,201)**		
	**Adalimumab (n = 669)**	**Etanercept (n = 628)**	**Infliximab (n = 171)**	**Adalimumab (n = 248)**	**Etanercept (n = 367)**	**Infliximab (n = 118)**	**Adalimumab (n = 917)**	**Etanercept (n = 995)**	**Infliximab (n = 289)**
Mean age^1^	57	58	58	56	55	55	56	57	56
Female^2^, %	88	88	89	87	89	86	88	89	88
Charlson Comorbidities Index^1,3^	1.62	1.69	1.52	1.49	1.35	1.42	1.58	1.56	1.48
1. Myocardial infarction	1.08%	2.20%	0.61%	0.43%	0.91%	1.89%	0.91%	1.74%	1.11%
2. Congestive heart failure	4.62%	4.06%	3.05%	4.33%	3.66%	1.89%	4.54%	3.92%	2.59%
3. Peripheral vascular disease	4.00%	6.60%	4.88%	3.90%	3.96%	5.66%	3.97%	5.66%	5.19%
4. Cerebrovascular disease	4.46%	3.72%	2.44%	1.73%	2.74%	1.89%	3.75%	3.37%	2.22%
5. Dementia	0.46%	0.34%	0.00%	0.00%	0.00%	0.00%	0.34%	0.22%	0.00%
6. Chronic pulmonary disease	14.46%	14.38%	9.15%	19.91%	14.33%	11.32%	15.89%	14.36%	10.00%
7. Peptic ulcer disease	2.92%	2.20%	2.44%	2.60%	1.83%	5.66%	2.84%	2.07%	3.70%
8. Mild liver disease	9.54%	9.64%	8.54%	8.66%	4.88%	9.43%	9.31%	7.94%	8.89%
9. Diabetes without chronic complication	10.15%	13.54%	9.15%	9.96%	8.54%	9.43%	10.10%	11.75%	9.26%
10. Diabetes with chronic complication	2.31%	4.91%	3.05%	2.16%	2.13%	4.72%	2.27%	3.92%	3.70%
11. Hemiplegia or paraplegia	0.92%	0.68%	0.00%	0.43%	0.61%	0.00%	0.79%	0.65%	0.00%
12. Renal disease	3.85%	4.40%	5.49%	1.73%	4.57%	0.94%	3.29%	4.46%	3.70%
13. Any malignancy. including lymphoma and leukaemia. except malignant neoplasm of skin	2.46%	4.23%	3.05%	1.30%	1.83%	2.83%	2.16%	3.37%	2.96%
14. Moderate or severe liver disease	0.00%	0.00%	0.00%	0.00%	0.30%	0.00%	0.00%	0.11%	0.00%
15. Metastatic solid tumour	0.31%	0.17%	0.00%	0.00%	0.00%	0.00%	0.23%	0.11%	0.00%
16. AIDS/HIV	0.00%	0.17%	0.00%	0.43%	0.00%	0.00%	0.11%	0.11%	0.00%
	F- and p-values								
	**Mean age**	**Gender**	**Charlson index**	**Mean age**	**Gender**	**Charlson index**	**Mean age**	**Gender**	**Charlson index**
Adalimumab vs. etanercept	2.6254	1.000	1.3998	1.679	0.957	2.205	0.7768	1.000	0.5637
Adalimumab vs. infliximab	1.5851	1.000	1.3518	1.455	1.000	0.803	0.1062	1.000	2.0666
Etanercept vs. infliximab	0.1165	1.000	2.2446	0.233	0.825	0.8645	0.4249	1.000	1.7001
	Tukey-Kramer	Bonferroni	Tukey-Kramer	Tukey-Kramer	Bonferroni	Tukey-Kramer	Tukey-Kramer	Bonferroni	Tukey-Kramer

^1^Tested with Tukey–Kramer test.

^2^Tested with Bonferroni method.

^3^Based on ICD-10 Coding Algorithms for Charlson Comorbidities by Quan et al. [16].

The Charlson Comorbidities Index was 1.62 for adalimumab naive patients, 1.69 for etanercept naive patients, and 1.52 for infliximab naive patients. For the TNF Inhibitor continuing patients the Charlson Comorbidities Index was 1.49 for adalimumab, 1.35 for etanercept, and 1.42 for infliximab. The Index can range between 0 and 33 with higher numbers indicating higher comorbidity. Hence, differences in mean terms as observed in the study populations do not seem to reflect clinically meaningful differences in terms of comorbidity. The means of the Charlson Comorbidities Index are not significantly different from each other (Tukey–Kramer test, p < 0.05). Table 1 shows the F-value Tukey–Kramer test and the p-value for the Bonferroni method.

### Persistence

**Figure 3:**
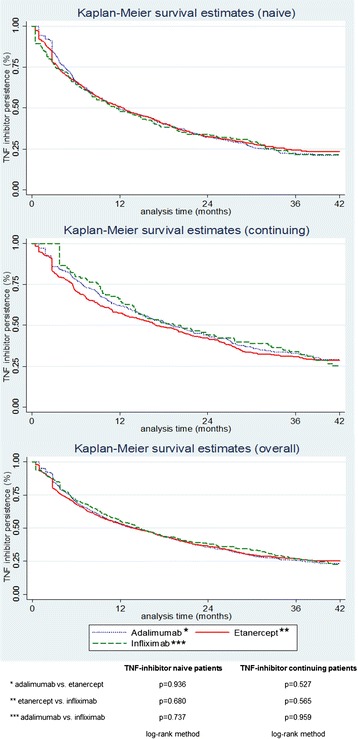
**Kaplan-Meier estimates for TNF inhibitor persistence over time.**

Figure 3 illustrates the Kaplan Meier estimation for TNF inhibitor naive and continuing patients during the follow-up period of 3.5 years (42 months). There were no significant differences in the persistence rates between adalimumab, etanercept and infliximab for TNF inhibitor naive patients (adalimumab vs. etanercept: p = 0.936 etanercept vs. infliximab: p = 0.680; adalimumab vs. infliximab: p = 0.737 performing the log-rank test). Furthermore, there were no significant differences for continuing patients (adalimumab vs. etanercept: p = 0.527; etanercept vs. infliximab: p = 0.565; adalimumab vs. infliximab: p = 0.959 performing the log-rank test). The persistence rate after one year for naive patients was 50.96% for etanercept, 49.95% for adalimumab, and 47.95% for infliximab. After two years the persistence rate was 32.19% for adalimumab, 32.81% for etanercept and 34.05% for infliximab. After year three, the persistence rate was 22.47% for adalimumab, 24.27% for etanercept and 21.49% for infliximab. The differences in persistence are apparently small. This is also shown in the graphs in the overall figure, which shows the naive and continuing patients data summed up.

For TNF inhibitor continuing patients the overall persistence rates after 1, 2 and 3 years were 62.10%, 43.87% and 32.88% for adalimumab; 57.49%, 42.20% and 30.95% for etanercept; and 66.10%, 44.07% and 33.90% for infliximab, respectively.

### Impact of comorbidities and other factors on persistence of TNF inhibitor therapy

Gender, medication and Charlson Comorbidities Index did not influence the persistence significantly. No significant hazard ratios (HR) were obtained in the Cox regression. For the continuing patients, age has a significant influence on persistence (p ≤ 0.005). Old age lowered the risk of discontinuation of TNF inhibitors (HR: 0.990, 95% CI: 0.983-0.997, p ≤ 0.005) (Table 2). Between naive and continuing patients a significant difference in persistence could be observed (HR: 1.296, 95% CI: 1.164-1.443, p = 0.000). Being a naive patient was associated with an earlier discontinuation of TNF inhibitors. Furthermore, all HR in Table 2 are close to 1.0, therefore, the impact is considered to be relatively low.

**Table 2:** **Cox proportional hazard analysis (95% CI) for persistence of TNF inhibitor**

	**HR**_**naive**_ **(n = 1,468)**	**HR**_**continuing**_ **(n = 733)**	**HR**_**overall**_ **(n = 2,201)**
Gender (0: male/1: female)	0.942 (0.780-1.138)	0.937 (0.723-1.215)	0.943 (0.810-1.099)
Age	0.999 (0.994-1.005)	0.990 (0.983-0.997)*	0.996 (0.992-1.000)
Medication (reference category: Infliximab)			
Etanercept	0.960 (0.788-1.170)	1.084 (0.850-1.381)	1.001 (0.865-1.175)
Adalimumab	0.956 (0.786-1.163)	1.023 (0.790-1.323)	0.983 (0.842-1.148)
Charlson Comorbidities Index	1.018 (0.968-1.071)	1.055 (0.974-1.143)	1.030 (0.987-1.075)
Naive	-	-	1.296 (1.164-1.443)**

*p ≤ 0.005.

**p = 0.000.

### Overall TNF inhibitor discontinuation and switch analysis

Over the whole observation period the number of patients with overall TNF inhibitor discontinuation, i.e. patients who receive no other TNF inhibitor prescription after a discontinuation of the first choice, is 24.22% in adalimumab naive patients (16.53% in adalimumab continuing patients), 19.59% in etanercept naive patients (8.17% in etanercept continuing), and 22.81% in infliximab naive patients (22.88% in infliximab continuing). When considering not only the index medication choice but all anti-TNF prescriptions, the overall per patient discontinuation rate was 64.51% for TNF inhibitor naive and 70.94% for TNF inhibitor continuing patients over the entire observation period.

### Dosage increase rates

Exceeding the labelled dosing in the time frame of one year after the index event in the TNF inhibitor naive patients occurred in 7.3% of adalimumab, 1.4% of etanercept and 17.2% of infliximab treated patients. Respective figures for the TNF inhibitor continuing patients were 5.8% in the adalimumab group, 1.1% in the etanercept group and 11.9% in the infliximab group. This reflects the current label of all three TNF inhibitors, as only adalimumab and infliximab allow for dose increases whereas etanercept has a stricter regime that cannot be adapted.

### Sensitivity analysis

For sensitivity analyses the gap between two consecutive TNF inhibitor prescriptions was set at 30 and 120 days. The average persistence rate for 30 days was 10.62% over all patients in the first year after the index event. The overall persistence rate was 14.35% for adalimumab naive, 9.30% for adalimumab continuing, 14.49% for etanercept naive and 10.35% for etanercept continuing patients.

With a gap cap set at 120 days the average persistence rate was 44.45%. The persistence rate for adalimumab continuing patients was 45.20%, 48.77% for etanercept and 37.29% for infliximab. The persistence for TNF inhibitor naive patients was 44.84% for adalimumab, 47.93% for etanercept and 42.69% for infliximab.

The number of patients with at least one hospitalization was also analysed within a sensitivity analysis, as these patients might have received a TNF inhibitor application during that time. The percentage of patients which had at least one hospitalization for any reason ranged from 72.88% in the infliximab TNF inhibitor continuing patients to a maximum of 82.51% of the adalimumab TNF inhibitor naive patients (Table 3). The percentage of patients with an inpatient stay in hospital related to the RA-disease ranged from 38.31% of the adalimumab TNF inhibitor continuing patients to a maximum of 46.18% in the etanercept TNF inhibitor naive patients. Even if days of inpatient stays were summed up to the permissible gap the persistence rate did not change significantly.

**Table 3:** **Hospitalization/inpatient treatment**

	**TNF inhibitor naive patients (n = 1,468)**			**TNF inhibitor continuing patients (n = 733)**		
	**Adalimumab (n = 669)**	**Etanercept (n = 628)**	**Infliximab (n = 171)**	**Adalimumab (n = 248)**	**Etanercept (n = 367)**	**Infliximab (n = 118)**
Numbers of patients with at least hospitalization for any reason (%)	522	498	126	190	278	86
	78.03%	79.30%	73.68%	76.61%	75.75%	72.88%
Numbers of patients with hospitalization related to RA	298	290	66	95	162	47
	44.54%	46.18%	38.60%	38.31%	44.14%	39.83%

## Discussion

This study was based on claims data of a major health insurance fund and evaluated the persistence and the dosage increase rates of TNF inhibitors in patients with RA. The strength of this study is that all results are based on real-world data representing the daily life treatment setting in Germany. In addition our study is the only study which considered all three TNF inhibitors (adalimumab, etanercept, and infliximab) over a longer observation period for Germany. To our knowledge it is the first approach which analysed persistence and dosing patterns for all patients independently by which medical professionals they were treated.

Several studies have evaluated TNF inhibitor persistence [8–14]. The findings of the current study are consistent with those of Cho et al. who found that there is no significant difference in persistence between etanercept and adalimumab [12]. Although these results are similar, Cho et al. show a higher persistence. After 12 month our results in persistence for continuing patients are quite similar to those of Jobanputra et al. (adalimumab 67,7% vs. 65.0% and etanercept 58,9% vs. 56,7%). After the second year our persistent decreased to 48,8% (adalimumab) and 42,8% etanercept whereas the results of Jobanputra et al. shows a higher persistence for adalimumab (58,3%) and similar for etanercept 43,3% [10]. Compared to the results of Carmona and Gomez-Reino, Kievit et al. and Zink et al. our persistence rates are similar, sometimes a bit lower than the other results [8,9,11,14]. A possible explanation for these results may be the gap length. Cho et al. permit a gap of 14 weeks for the persistent calculation. The gap of 98 days is considerably higher compared to the 60 days in our study. Something similar applies to the studies of Kievit et al. and Du Pan et al.. Both studies have a higher gap definition (3 months respectively 6 month) and state also a higher persistence after the first year [9,13]. A few studies do not state a definition of interruption [8,10,11,14]. That inconsistency may be due to the database because these studies based on clinical datasets or register. In this data discontinuation dates were set externally e.g. from an involved physician.

The higher gap definition could be an explanation for the founding in differences in the study of Fisher et al.. Moreover the study population was restricted to patients who were under 65 years of age, contrarily to our study which includes all patients older than 18 years. They analysed dosing patterns for all three TNF inhibitors in the United States with managed care data for four years [21]. This study found significant differences in the treatment persistence rates between adalimumab, etanercept and infliximab. In our study, there were no significant differences in persistence between the three TNF inhibitors. Carmona et al. did not analyse differences by medication but in persistence at one, two, and three years by diagnosis. They detected that there was a significantly greater persistence in spondylarthritis than in RA. The current study excluded spondylarthritis from the analyses.

Given the linear relationship between dosage and costs, dosage increases in clinical practice may have significant cost implications for patients and payers [22]. A recently published RA treatment algorithm advocates shortening the dosing interval of adalimumab or increasing the dose or shortening the dosing interval of infliximab in patients with an inadequate response prior to switching to another TNF inhibitor [23]. Therefore, it is important to understand the dosing regimens used for TNF inhibitors in clinical practice.

Dosage increase rates identified for adalimumab and infliximab in the current study were similar to those reported by other studies. Upward dosage adjustment of infliximab in patients with RA has been associated with increases of 30–50% in medication costs in recent studies [24,25]. For instance, Harrison et al. reported that among naive and continuing patients, dose increases from the first to the last prescription were more likely to occur for infliximab (26% and 24%, respectively) than adalimumab (10% and 9%, respectively) or etanercept (1% and 3%, respectively) [22]. In the study of Wu et al. all treatments had similar dose reduction rates, but terms of dose-increase rates infliximab had the highest with 28.3% compared to adalimumab (8.7%) and etanercept (6.9%) from the payer perspective [17]. While etanercept had lower dose escalation rates than adalimumab, this could probably be explained by labelling which advises against dose escalation. Berger et al. and Agarwal et al. analysed only the pattern of infliximab utilization [26,27]. The mean dose increase by Berger et al. over 12 months was 36%. One-half of study population had their dose of infliximab increased by equal to or greater than 30% between the initial and final infusions; one-third had their dose increased by equal to or greater than 50% [26]. In the study of Agarwal et al. 126 patients (68.8% of the study population) had a treatment escalation (19.8% patients with a dose increase, 27.8% patients with a decrease in the interval, and 52.4% patients with both) [27]. While etanercept had lower dose escalation rates than adalimumab, this is consistent with their labelling which advises against dose escalation.

All the previous mentioned studies have evaluated TNF inhibitor dose escalation, but there is no standard analytic method for the calculation of dose escalation. This fact could explain most of the differences across the studies [28,29].

This study has some limitations. Compared to a clinical trial there is no direct clinical data input (e.g. disease activity, severity grades of a disease, symptom scores, clinical test results, quality of life data) as health insurances in Germany are prohibited by federal law to gain knowledge of any clinical data from their customers. Furthermore, there are no clinical outcome data available, so the reason or impact for the discontinuation or dose increase cannot be assessed. In general, reasons for discontinuation include remission of the disease, a lack of patient compliance or a change in therapy due to side effects. German claims data contains no information about medications administered during an inpatient episode as hospitals receive no additional payments on top of the respective DRG for the supply of medications. However, the sensitivity analysis showed that even if the days of inpatient stays were added to the permissible gap as per definition in the analysis persistence rates did not change significantly. Another limitation is present for the TNF inhibitor continuing patients. These patients per definition had at least one TNF inhibitor prescription during the baseline period and at least one claim in the follow-up period. However, it cannot be assessed when they initially might have started their TNF inhibitor therapy before the baseline period and for how long they already have been on TNF inhibitor medication. Interpretation of reason for the different persistent and dosing patterns is challenging because there are no information about the disease duration in the claims data. By law health insurance funds are allowed to save the data for the insured persons for only maximum of five years. Therefore, we had only this time period for the observation and analysis. This is a limitation of our study because this missing information about disease duration and the severity grades of a disease could influence the rate of persistence. Another confounding variable could be the side effects of medication. In the data we do not see the intolerance to pharmaceutical ingredients or medications, but this could decrease the rate of persistence. Also the effects of medication, if one medication has no effect for the patient, as well as preferences of the physician and patient to one specific medication could influence the rates.

The dosing calculations were based on filed claims paid by the health insurance. Thus, the results may not reflect the actual amount of infliximab administrated to the patient and may over- or underestimate the dosing for any infusion. For example, a patient that in reality increased his dosage from 1.2 to 1.8 vials would have appeared to have a stable dose of two vials if the interval between infusions remained the same, whereas a patient increasing from 1.6 to 2.2 vials would have been considered to move from 2 to 3 vials in terms of claims made to the health insurance. Furthermore, the present study did not separate between combination therapy and monotherapy. We also excluded patients with other inflammatory disease like Crohn´s disease, because this indication is treated with TNF inhibitors as well. This will probably affect the results.

The results of the current study may be generalizable to other patients with RA, but not be representative of the entire German population. E.g. the rates of hospital admissions in this population is very high (up to 82%) even the persistence rate did not change significantly although the inpatient stays were summed up to the allowable gap. One reason could be the population itself. We did not restrict to high age of the patient. Moreover the average duration of stays was less than 10 days. So the rates are very high but the duration is quite short. Additionally, in context of the results there is no reason to expect that hospitalisation is disproportionately distributed among the three study cohorts.

## Conclusions

In summary, the results of this study provide insights into dosing patterns and persistence of TNF inhibiting agents for treatment of RA in Germany. The findings reflect the real-life use of TNF inhibitors without the limitations of a clinical trial and allow for a head to head comparison although the inherent limitations of retrospective claims data analyses do not allow for causal conclusions. Furthermore, persistence to treatment can improve the medical outcome and reduce costs. In addition the results of this paper can be used as input factor for models, for example in cost-effectiveness analyses.
